# A critical view on using “life not worth living” in the bioethics of assisted reproduction

**DOI:** 10.1007/s11019-023-10191-7

**Published:** 2024-02-16

**Authors:** Agnes Elisabeth Kandlbinder

**Affiliations:** PhD Researcher, URPP Human Reproduction Reloaded, High-Risk Sub-Project 4, Department of Philosophy, University of Zurich, Zollikerstrasse 115, 8008 Zurich, Switzerland

**Keywords:** CRISPR, Germline genome editing, Preimplantation genetic diagnosis, Assisted reproductive technologies, Beginning-of-life, Quality-of-life, Disability

## Abstract

This paper critically engages with how *life not worth living* (LNWL) and cognate concepts are used in the field of beginning-of-life bioethics as the basis of arguments for morally requiring the application of preimplantation genetic diagnosis (PGD) and/or germline genome editing (GGE). It is argued that an objective conceptualization of LNWL is largely too unreliable in beginning-of-life cases for deriving decisive normative reasons that would constitute a moral duty on the part of intending parents. Subjective frameworks are found to be more suitable to determine LNWL, but they are not accessible in beginning-of-life cases because there is no subject yet. Conceptual and sociopolitical problems are additionally pointed out regarding the common usage of *clear case* exemplars. The paper concludes that a moral requirement for the usage of PGD and GGE cannot be derived from the conceptual base of LNWL, as strong reasons that can be reliably determined are required to limit reproductive freedom on moral grounds. Educated predictions on prospective well-being might still be useful regarding the determination of moral permissibility of PGD and/or GGE. It is suggested that due to the high significance of subjective experience in the normativity of beginning-of-life bioethics, the discipline is called to more actively realize the inclusion of people with disabilities. This regards for instance research design, citation practices, and language choices to increase the accessibility of societal debates on the reproductive ethics of genetic technologies.

## Introduction and preliminaries

The recent development of the CRISPR/Cas9 method has produced new outlooks for a possible future of applying germline genome editing (GGE) in human reproduction (see Jinek et al 2012; Doudna and Charpentier 2014). A hotly debated question in current bioethics is whether an intervention with GGE in the process of in-vitro-fertilization could be justified if risks in the application could be sufficiently limited (see Nuffield Council on Bioethics 2018; Deutscher Ethikrat 2019). Many of the previously made arguments about morally permissible or even required uses of preimplantation genetic diagnosis (PGD) are often reworked and can partly be appropriated when discussing the technology of GGE.^1^ Various authors in bioethics are thus debating parental responsibilities to use those technologies to avoid bringing a child with a certain genome into existence, or to create a child with a different genome than would have otherwise been born.^2^ Some have prominently suggested that intending parents are morally best advised to use assisted reproductive technologies (ARTs) to create ‘the best possible children’ that will presumably lead a future life with the ‘greatest expected personal well-being’ through means of ART (see Buchanan et al. 2000; Harris 2007). Some conceptualize this in terms of a moral duty or imperative that parents have towards their children to provide them with some amount of minimally expected welfare (see Purdy 1996; Archard 2004), or maybe even the best possible welfare (see Munson and David 1992; Savulescu 2001; Gyngell et al. 2019). Even careful positions (see Glover 2006), categorically critical authors (see Habermas 2003), or defenders of a personalist view (see Gavaghan 2007) maintain a caveat for either permissibility of genetic interventions or a corresponding parental obligation in assisted reproduction for the avoidance of *very serious* conditions.^3^

An axiom that is frequently present in these positions is a *life not worth living*^4^ (henceforth: LNWL) or cognate concepts such as a “minimally satisfying life” (Purdy 1996), “minimally acceptable life” (Archard 2004, p.406), or “minimally decent existence” (Steinbock 1986, p.19). Cognate concepts rest on LNWL in the sense that they also define a concept of minimal life-quality that triggers the same normative implication. Their shared normative function is as follows: If such a (future) life of below-minimal quality is determined to be (very likely) the case, it provides “moral reasons to not to cause that person to exist, and indeed a reason to prevent that person from existing” (McMahan 2009, p.49). Since LNWL and cognate concepts are presented in structurally similar ways, and therefore share many of the same conceptual problems, they are analyzed together here.^5^ Some authors argue that prospective parents have special duties if their prospective child’s existence can be classified as a LNWL. These moral duties include a negative duty to refrain from reproducing when a LNWL would (likely) be the outcome, or alternatively, a positive duty to use reproductive technologies to ensure that a LNWL will not be the result of one’s enactment of reproductive freedom. More precisely, this means either genetically testing an embryo and discarding it if certain genetic features are present, or genetically modifying the feature(s) in question before inducing a pregnancy. Working with LNWL as an axiom and formulating arguments subsequently derived from the concept to make moral claims about reproductive decisions can be said to mark a widespread consensus in bioethics (Ranisch 2017, p.364) and has been termed “bioethics orthodoxy” by Wilkinson (2010, p.97).

This article examines how LNWL and cognate concepts are used and operationalized within some influential bioethical positions.^6^ It is argued that an objective conceptualization of LNWL is largely too unreliable in beginning-of-life cases to provide us with normative reasons decisive enough to constitute moral duties of intending parents. This paper defends a subsidiary claim that LNWL judgments can only be reasonably made within a subjective framework, which is not accessible in beginning-of-life as there is no subject yet. Additionally, sociocultural considerations are supplementarily brought forward regarding the often-underrepresented perspectives of people with disabilities and their carers. It is suggested that of corresponding debates can be improved by more effectively incorporating their subjectivities in normative research.

The presented argument presupposes the value of reproductive autonomy to underpin a liberty right, whereof strong reasons are required to justify restrictions. This is in line with the practical reality of a broad institutional and societal consensus on the value and primacy of reproductive autonomy in liberal-democratic societies. To uphold the foundations upon which such liberal-democratic societies are built, individual actors (e.g. ethicists, medical professionals, intending parents) are called to reflect on their positionality, foster awareness and critical engagement with social and cultural conditioning, and enact tolerance for differences in opinions and experiences. This ensures consistency of ‘reproductive autonomy’ in both theory and quotidian by avoiding biases to appear ontological and morally universal. This paper adds to this larger endeavor by critically investigating the axiomatic concept of LNWL in the bioethics of assisted reproduction.

In contrast to a previous argument brought forward by Roberto Fumagalli for “the elimination of the concept of life worth living from philosophical vocabulary” (Fumagalli 2018, p.769), this paper only criticizes the way LNWL is commonly used in the context of beginning-of-life. I grant that LNWL could still be useful in other contexts, such as end-of-life ethics.^7^ I consequently do not intend to make the statement that every life is inherently worth living. I also do not propose that there are no reasons that could justify possible applications of PGD or GGE.

In the literature, the concept of LNWL also frequently arises in the context of abortion as well as prenatal genetic diagnosis for selective abortion. Some of the authors I critique (e.g. Purdy 1996; Buchanan et al. 2000) include using the combination of prenatal genetic diagnosis and selective abortion in their scope of parental responsibility to avoid LNWL. I will not be addressing these procedures here, as the normative situation of an existing pregnancy differs significantly from the one of pre-implantation genetic testing/editing. I want to state upfront that I reject any conclusion that attempts to derive a categorical opposition to abortion or even selective abortion from my critique. Abortion care is fundamental to reproductive justice (see Ross and Solinger 2017) and all pregnant people should have safe access to it. I also do not support the view that practices of performing PGD, selective abortions, or hypothetically GGE, inherently and primarily necessitate a LNWL judgment on behalf of parents and/or medical practitioners. This would be inconsistent with the qualitative data we have on the matter, that point to reasons, considerations, and affective responses of people involved in such situations being vastly more complex than that (see e.g. Lie et al. 2008; Järvholm et al. 2014; Gammeltoft 2014; Purcell et al. 2017; Beynon-Jones 2017; Altshuler et al. 2017; see also Böcker 2022).

The formulation of moral suggestions or even duties has the potential to influence interdisciplinary academic (e.g. legal studies) as well as public and political debates on ARTs.^8^ By co-shaping discursive context, prevailing attitudes in beginning-of-life bioethics might thus affect policy-making endeavors (e.g. through ethics boards) or sway individual decisions indirectly. This paper makes no claim on the empirical question of whether genetic counseling in clinical contexts is directly impacted by LNWL, hence whether the moral underpinnings of these arguments are actively being imposed on intending parents. Whether the concept of LNWL is relevant for prenatal decision-making by prospective parents themselves is ambiguous in qualitative research on the matter. A number of studies suggest that parents do take their prospective child’s level of quality of life into account during prenatal decision-making (see e.g. Bell and Stoneman 2000; Sandelowski and Barroso 2005; Korenromp et al. 2007; Gammeltoft 2014; Blakeley et al. 2019). Uncontroversially, I shall argue later that they are morally justified to do so. However, the qualitative data is not conclusive on LNWL as conceptualized in the bioethical literature being a helpful tool in practice for parents’ decision-making. Some findings suggest that medical providers’ normative assessments of certain conditions as for instance ‘incompatible with life’ can be experienced as unhelpful by parents (see Koogler et al. 2003; Janvier et al. 2012; Guon et al. 2014).

In the following analysis, the scrutinized LNWL judgments are not the ones of prospective parents or medical practitioners in these situations, but of bioethicists using the concept to develop their positions. Thus, the conceptual argument here is not primarily concerned with any concrete, real life decision-making cases such as those faced by prospective parents who receive a prenatal diagnosis. Yet, it is important to acknowledge that there are discursive interrelations between those contexts. Bioethics as a generative discursive force of creating authoritative knowledge arguably has a certain power to influence attitudes, legislation, and practice on reproductive technologies. Likewise, individual decision-makers additively contribute to the creation and preservation of cultural narratives about what a good life is, what is normal, what constitutes a tragedy, etc. Yet, it would be too simplistic to paint either of these influences as one-sided or causal. Within this complex discursive context, this paper investigates a part of the bioethical debate about beginning-of-life that aims to derive more general normative statements through the deliberate abstraction of real cases.

## Whose *best judgment*? On LNWL determinability and why it is not given in beginning-of-life

This section examines in detail how LNWL shows up in bioethical literature on reproductive decision-making, and why it fails to be sufficiently determinable in beginning-of-life to substantiate claims of reproductive moral obligations.

Section “Proposals in the literature on how LNWL can be identified” presents the three ways that LNWL is identified in beginning-of-life literature:

- *Objective quality evaluation,*
- *Subjective quality evaluation,*
- *Clear case exemplars.*

I subsequently argue in Sect. “Why LNWL is insufficiently determinable in beginning-of-life” that the above accounts, individually as well as combined, cannot determine when LNWL can be said to be the case in beginning-of-life. This conclusion is founded on a principal critique of objective approaches to determine LNWL as well as the problem that subjective approaches are not accessible in beginning-of-life. This leads to the derivation of my two independent main claims, namely that (1) objective LNWL is a problematic concept, and (2) moral obligations to apply PGD and/or GGE that are grounded in avoiding the creation of a LNWL are not justified. The latter claim assumes that strong reasons that can be reliably determined in a particular case scenario are required to limit reproductive freedom on moral grounds. I additionally suggest some sociocultural considerations on why the common presentation of LNWL through *clear case* exemplars might act to exclude people with disabilities from the discussion.

### Proposals in the literature on how LNWL can be identified

The three following approaches show up in the beginning-of-life literature implementing LNWL. They are proposed most often in a combined, interconnected manner to present what LNWL is and how it is identified. The division into three accounts here is solely for analytic purposes. Taken together, these accounts exhaustively encapsulate how LNWL is conceptualized in beginning-of-life and thus ARTs-related bioethics. Detailed examinations of the concept itself are rare.^9^ LNWL usually serves as an implement in building a larger argument of parental responsibility. Every LNWL conception is necessarily built on an underlying framework of a theory of *well-being/the good life*. Most often, the commitment of LNWL conceptions to a specific framework of *well-being* is not transparent in the literature. Thus, I have assigned the common philosophical frameworks of *well-being* to the accounts for structural purposes.

- Objective quality evaluation

This account suggests establishing objectivized (comparative or structural) evaluations of life quality. The corresponding frameworks of *well-being* include *objective list* theories^10^ as well as *informed preference* approaches.^11^ While the former evaluates prospective life quality based on predetermined criteria, the latter assumes a rational desire on the part of the prospective person to live (or not live) a certain kind of life under *preferable* conditions. The evaluation, depending on the author, can be done in a few different ways that also overlap at times. One is the so-called *threshold* view: Below a certain threshold or zone, termed by Glover (2006) as “the zero-line” (p.52) or a less clear-cut “grey area” (p.58), the level of life quality is identified to be so low as to plausibly constitute LNWL. Relatedly, some authors take a *minimal conditions* view that determines LNWL to be the case when certain basic objectivized conditions cannot be met (see e.g. Purdy 1996; Archard 2004; Steinbock 2009). This is sometimes correlated with a *worse-than-nonexistence* comparison (see also Benatar 2007) that describes LNWL as follows:^12^

[T]here are possible cases of what is standardly termed ‘wrongful life’ or ‘life not worth living’, situations in which the person’s quality of life is *so bad* that they would be ‘better off dead’ or ‘better off not existing’. […] it is wrong (in the absence of a special justification) to create a child that will have a sub-zero quality of life. (Wilkinson 2010, p.97, italics added)

Buchanan et al. (2000) also formulate:”an infant’s or child’s quality of life is so bad that he or she would have been better off never having been born at all” (p.225).

Another common approach is a *balancing* view, implementing a harm/benefit calculation of life quality that identifies a LNWL if it “would contain such a balance of harms over benefits [for the child] that its life would constitute a ‘harm on balance’” (Gavaghan 2007, p.97). McMahan (2009) summarizes it as follows: “[A] life that is ‘worth not living’ [is] a life in which the intrinsically bad states outweigh the good [which] provides a moral reason not to cause that person to exist, and indeed a reason to prevent that person from existing” (p.49). Bennett (2009) also mirrors this approach: “extreme suffering completely outweighs any positive experiences” (p.266). A *balancing* view is also presented by Buchanan et al. (2000):

[P]reventing wrongful life by abortion is also a matter of protecting an innocent, defenseless third party from the great wrong of being born with a life so dominated by suffering and without compensating goods that it is a life not worth living. (Buchanan et al. 2000, p.239f.)

Similarly, we find this proposal in Gavaghan (2007), who simultaneously adopts the *worse than nonexistence* comparison:

[T]hose cases where the child’s life is so awful that we can actually deem it worse than non-existence, or worse than nothing (WTN). Such a judgment could be made where the child’s most important interests are doomed from the outset – such as its interest in avoiding intolerable suffering – while at the same time no corresponding interests could be furthered. (p.92)

While authors differ in their methodologies on how LNWL-determining aspects are weighed, all these views can be plausibly unified in their commonality to determine LNWL in beginning-of-life through external assessments of expected life quality. While a fledged-out list of criteria is often lacking (exceptions being Archard 2004; Glover 1977, 2006), these assessments are typically made with recourse to certain conditions such as low life expectancy, high levels of pain and duration of pain, high levels of suffering, and more.

(b) Subjective quality evaluation

This approach establishes a LNWL to be the case when it is *subjectively evaluated* as such. The prospective person would thus *prefer* being dead over continuing their existence, or *prefer* not having come into existence at all.^13^ The corresponding theories of well-being are *hedonistic* approaches as well as pure *desire fulfillment* views. Hedonism, on its simplest account, assesses well-being based on a balancing of “pleasure over pain” (Crisp 2021; see also Bentham 1789[1996]), while desire fulfillment evaluates well-being on the degree of (personal) desire satisfaction (see Heathwood 2016).^14^ Methodologically, a subjective LNWL judgment is mostly argued through some form of *balancing* or *threshold* approach, as presented previously.

Purdy (1996) defines people leading a LNWL as being “so miserable that they wish they were dead” (p.45). Singer (2011[1980]) also includes this element when illustrating a LNWL judgment as “the life of an infant will be so miserable as to not be worth living, from the internal perspective of the being who will lead that life” (p.162). As this shows, subjectivist approaches ground themselves in the *actual subjective desire and preference* of the people in question. Gavaghan (2007) uses this approach, combined with elements of objective evaluation, in the following quote: “a life of such wretched quality that, from the subjective perspective of the child itself, it would have been better to never have been born.” (p.92).

In this account, the person’s subjective *desire* or *preference* to not come into existence or the absence of (enough) personally pleasurable mental states/fulfilled desires is operationalized in order to determine whether LNWL can be said to be the case.

(c) Clear case exemplars

This complementary proposal instrumentalizes the selective narrative presentation of what is referred to as *clear cases*: genetic conditions that are thought to represent obvious illustrations of LNWL. Counter to the two previous proposals, clear case exemplars are not linked to objectivist or subjectivist frameworks of well-being. Instead, they are aimed towards facilitating *intersubjectivity* (see Husserl 1999) between readers and the (prospective) persons in question, meaning “the interchange of thoughts and feelings, both conscious and unconscious, between two persons or ‘subjects,’ as facilitated by empathy” (Cooper-White 2014, p.882). The purpose is to showcase that certain genetic traits will determine someone’s subjective state as LNWL by readers’ empathic and intelligible intuition.

The *clear case* conditions are often similar across different authors. Singer (2011[1980]) uses Tay-Sachs (see p.161f.), Purdy (1996) Corea Huntington’s (p.42ff.) as an example. Gavaghan (2007, p.92f.) and Glover (2006, p.59f.) refer to Lesch-Nyhan syndrome. On the latter, Gavaghan (2007) cites the following passage by philosopher Kitcher (1996):

[A]n allele on their single X chromosome causes boys to suffer mental retardation and extreme pains of the type associated with gout. Yet perhaps the most disturbing feature of the condition is an apparently irresistible urge to self-mutilation – the boys chew their lips and the tips of their fingers until they are raw and bleeding.” (Kitcher 1996, p.82)

Purdy (1996) quotes *The Merk Manual* (1972) on Huntington’s at length:

Onset is insidious. Personality changes (obstinacy, moodiness, lack of initiative) frequently antedate or accompany the involuntary choreic movements. These usually appear first in the face, neck, and arms, and are jerky, irregular, and stretching in character. Contractions of the facial muscles result in grimaces, those of the respiratory muscles, lips and tongue lead to hesitating, explosive speech. Irregular movements of the trunk are present; the gait is shuffling and dancing. Tendon reflexes are increased…Some patients display a fatuous euphoria; others are spiteful, irascible, destructive, and violent. Paranoid reactions are common. Poverty of thought and impairment of attention, memory, and judgment occur. As the disease progresses, walking becomes impossible, swallowing difficult, and dementia profound. Suicide is not uncommon. (pp.1363, 1346)

The narrations of clear cases usually focus on physiological symptoms and psychological consequences, which are cited primarily from medical manuals. They evoke a vivid image resulting in uncomfortable affective responses such as disgust, fear, or pity. Conclusions are usually phrased similar to Singer (2011[1980]): “This seems to be a life that can be reasonably judged not to be worth living” (p.162).

This approach thereby aims to establish intersubjective plausibility of when LNWL can be said to definitively be the case.

### Why LNWL is insufficiently determinable in beginning-of-life

I now turn to argue why the proposals outlined in Sect. “Proposals in the literature on how LNWL can be identified” are altogether insufficient to describe when LNWL can be said to be the case in beginning-of-life. It is thus argued that LNWL fails to be satisfactorily determinable to offer decisive reasons that would justify moral duties to apply PGD and/or GGE in these cases. This rests on a principal critique of objective quality evaluations of prospective well-being (Sect. “Principal problems with objective quality evaluation to determine LNWL”) as well as two subsidiary critiques of the lack of access to subjective perspective in beginning-of-life cases (Sect. “Inaccessibility of subjective quality evaluation to determine LNWL”) as well as bias in intersubjective assessments (Sect. “Bias in clear case exemplars to illustrate LNWL”).

#### Principal problems with objective quality evaluation to determine LNWL

This section provides a critique of objective approaches to LNWL, or, more precisely, it rejects a subset of objective approaches with no regard for how subjective perspective is necessary to access objective goods for evaluation. Said objective approaches are deemed unreliable in beginning-of-life cases. I criticize an objective quality evaluation account on the assumption that solely using objective criteria could be epistemologically sufficiently suited to make a reliable quality-of-life-judgment in the first place. This is because it assumes that there are reasons to life being worth living that apply to everyone equally. Such objectivity of quality-of-life might not exist to the extent needed to justify deriving (especially positive) moral duties.

Critiques of this kind have been long brought up and well theorized by a significant amount of intersectional feminist and disability rights scholarship.^15^ It is argued that the missing reflexivity of researcher positionality inherent to objective and positivist approaches leads to isolated and therefore macrosocially incorrect judgments. This is especially the case if *ableist*^16^ bias is present, e.g. by presenting objective criteria solely as results of a disabled person’s bodily condition, failing to acknowledge the role of equally objective environmental barriers regarding their suffering. Objective assessments could be done not only with recourse to physiological symptoms, ergo locating the problem of disability only within the disabled body, but with equal attention to contextual circumstances and environmental factors, as suggested by advocates who have been rightfully critiquing the underlying *medical model of disability* for decades.^17^ It might be correct to assume that some physical and mental states objectively lower life quality (pain, having to undergo surgeries, etc.). Yet, operationalizing a corresponding, normatively relevant threshold of *too low* will not only overgeneralize people’s lived experiences by necessity, but will also be difficult to objectivize.

Consider the example of *suffering*. While much of the literature in the field of nursing acknowledges some link between pain and suffering (see e.g. Beecher 1957; Davis 1981; Cassell 1982), the same observational research of clinical practice identifies this link as less straightforward than it might intuitively appear: “There is an obvious relationship between the extent of pain and/or psychological distress and suffering. However, clinical observation suggests that focusing on this relationship is simplistic” (Kahn and Steeves 1986, p.625; see also Beecher 1959). Authors point to the complications of pain and suffering being both individually and culturally mediated (see Zborowski 1969; Petrie 1978), as well as suffering not merely being a response to pain, but equally occurring in anticipation of pain (see Copp 1974) or even in the complete absence of physical pain (Kahn and Steeves 1986, p.626). These experiential differences are further emphasized given the subjective component of suffering as a personal response and meaning given to pain (see Sarano 1970; Cassell 1982). While this does not mean that pain and suffering are irrelevant for life-quality evaluation, it complicates the determinability of how these factors will influence a future person’s experience of their life with a genetic variant. I argue that the inextricability of subjective perspective in determining a state of pain as one of suffering makes it impossible to apply a purely objective evaluation here.

Another example is the more easily objectivized state of *dependency*. Being substantially dependent on the assistance of others is a reason for some people in this condition to wish to end their lives. The 2021 data summary of the *Oregon Death with Dignity Act* (2022) indicates “loss of autonomy” (p.7), “[a]s in previous years” (p.7), as the most common reason for why end-of-life care patients wished to be assisted to die, with 93% of patients reporting it. However, it would be incorrect to therefore infer that this applies to all people who live under objectively similar conditions. Some people with disabilities who equally require significant personal assistance do not evaluate their dependency in this way at all. On the contrary, they report to view it as giving them reasons to relate to their environment and people in their lives in a deeply connected way (see Shildrick and Price 1998, 2002; Mingus 2017). Advocates of the disability justice movement have coined the concept of *interdependence* in this context (see Berne 2015), to emphasize on the one hand, how all (human and non-human) beings are constantly existing in a state of being dependent on one another, and on the other hand, to characterize this way of relating as inherently valuable by creating relationships built on vulnerability, intimacy, empathy, and care (see Mingus 2017; Piepzna-Samarasinha 2018; Aguayo-Krauthausen 2014). This is not to say that we should assume a homogenously positive interpretation of disability by affected persons. The point is rather to question any assumption of experiential homogeneity in this regard. Given that lived experiences of disability are so heterogeneous, it seems puzzling to assume that we could allocate a specific objective value judgment to e.g. the state of *dependency*.^18^

Adjacent to this is the problem of the common operationalization of objectivized harm/benefit *balances* being insufficient to establish LNWL judgments, an issue analogously shared by *threshold* approaches. Some people might believe it to be rather unintuitive to think that a life, as long as it still contains one or more beneficial aspects, could be deemed not worth living simply by minority calculation.^19^ The identification of LNWL as a life with “such a balance of harms over benefits that [it] would constitute a ‘harm on balance’” (Gavaghan 2007, p.97) is therefore not exhaustive. Whether people evaluate their lives as worth living or not is not only a matter of quantitative and qualitative harms over benefits, but also a matter of personal value emphasis. It is possible that statement (a) as well as statement (b) about person x is true:

- x’s life contains a lot of objective harms that detract significantly from their well-being.
- x experiences their life as worth living.

This also applies vice versa.

To illustrate this, people do not think of their level of life quality as objective harm/benefit balances, but rather evaluate according to personal weightings of certain aspects in their lives. In a life containing a majority of objectively benefitting aspects (such as meaningful connection with others, furthering of personal projects, etc.), a person could still evaluate the presence of a particular aspect as making their life not worth living (e.g. an unfulfilled wish for a romantic partner). Analogously, a person could still evaluate a life of overall more objectively harmful than beneficial aspects as worth living if a particular benefitting aspect to them was still given (e.g. the possibility of a certain artistic expression).

Another problem with many objective quality evaluation accounts is a circular reasoning around LNWL’s normative implication, instead of providing “precise and plausible referents for LWL” (Fumagalli 2018, p.782). Indicated objectivized criteria for a LNWL such as extreme suffering and low life expectancy usually remain too vague to constitute analytically sharp objective criteria. Clarification of which prudential goods cannot be obtained in a LNWL is often lacking.^20^ The normative implication is commonly set as the starting and end point of objective quality evaluation; all passages cited above (Sect. “Proposals in the literature on how LNWL can be identified” (a)) revolve around: *It is wrong to cause LNWL because LNWL constitutes a normatively significant harm*. Yet, while this is an explication of the normative force of LNWL, it still does not give any information about when and why LNWL can be said to be the case. The reasoning does not go far enough to detail what the base conditions for the normative outcome^21^ actually are, and when respective duties would be triggered.

Archard (2004) has proposed to overcome this shortcoming by suggesting the *United Nations Convention on the Rights of the Child* as a useful reference list, defining “the threshold of a minimally acceptable life as one in which the child has the reasonable prospect of enjoying a good number of these rights” (p.406). Glover (2006) equally provides some conditions for *a good life*:

[F]ood and shelter, protection and kindness are among the minimal conditions of a good life a child is owed […] [W]hat we owe to our children has to do with their having good lives […] There are obvious physical needs: food and drink, shelter and warmth, clothes, and medical care. They have non-physical needs too: love and warmth, security, the chance to make friends, stimulus, being talked to and being listened to, and the chance to develop their talents and themselves. (p.51)

These *minimal conditions* approaches surely have the advantage of accounting better for personal value emphases, such as a child’s possibility to develop their own interests and talents. Yet, the methodological shortcomings of these approaches become equally apparent: (a) With regard to GGE and PGD; most of the conditions proposed by both the *Rights of the Child* as well as Glover’s list of *a good life* are mostly unrelated to genetic traits. Whether or not they can be fulfilled is primarily determined by the quality of available social and institutional support systems, rather than instead of the presence/absence of a congenital health condition. Most of the articles in the *UN Convention of the Rights of the Child* start with the phrase ‘State parties shall…’, which also indicates that these conditions are subject to state responsibility and do not relate to parental reproductive duties.

And (b); such reference lists are often unsuitable to serve as truly workable minimal thresholds. They mostly fall back on rather describing aspirational living conditions; it is hard to argue that a lack of one or more particular minimal conditions would already constitute a LNWL, and vice versa. If e.g. Archard’s (2004) proposal were indeed a minimal threshold, the startling consequence would be that a good number of people, e.g. those living in countries that do not provide the fulfillment of a good number of the *Rights of the Child*, or people of low socioeconomic status and/or otherwise disadvantaged people, would have a moral obligation to not reproduce. In Steinbock’s (2009) words: “Instead of seeing the Rights of the Child as a minimal condition for morally permissible procreation, we should see it for what it is: an ideal” (p.167).

Further, I suggest that even if objective quality evaluations were committed to adopting a sound objective list framework of well-being, referencing clear criteria and thresholds to judge when a normative outcome is triggered accordingly, it would still not be enough to allow for the axiological conclusions that would support a value-of-life judgment. With this reasoning, I am sympathetic to Fumagalli’s (2018) analogous view that an evaluation of LNWL could not even be convincingly achieved by a hypothetically *ideal and fully informed caretaker* (see Fumagalli 2018, p.781f.; see also Smuts 2013, 2014), or *rational proxy chooser* (see Feinberg 1986, p.163ff.). Fumagalli’s (2018) critique goes as follows:

[E]ven if we knew all the facts that putatively determine whether a person’s life is [or is going to be] worth living, this *factual* knowledge would not *per se* provide informative *axiological* insights as to whether such life is worth living. […] [I]n the absence of a theory of how all facts that putatively determine whether a person’s life is worth living combine […], the Ideal Caretaker Criterion lacks the resources to ground informative LWL judgments. Conversely, once the criterion is supplemented with such theory, then the criterion itself becomes dispensable. (p.782, italics in original)

This problematizes the different philosophical dimensions that LNWL judgments claim to combine to fulfill their epistemic purpose. That is, a normative dimension (judging something as being better or worse relative to a standard), a pragmatic dimension (enabling taking satisfactory decisions), as well as an axiological one (providing moral grounding of decisions through value judgments). The pitfall that LNWL falls into lies in tautologically simplifying the axiological dimension for the sake of the pragmatic dimension: *a life can be decidedly stated not worth living (pragmatic) if it is so bad (normative) as to not be worth living (axiological)*. This, however, does not take the axiological dimension seriously in its own right. This shortcoming of LNWL conceptualization can also not be simply resolved by combining objective approaches with supplementary subjective criteria and/or *clear case* exemplars, as the theoretical grounding for the intended axiological conclusion remains absent.

This critique does not imply, however, that nothing at all can be said about future people’s well-being, or the bettering/worsening effects of present actions on it. Yet, such judgments will by necessity have a degree of unreliability and speculation to them. They will thus rather constitute assumptions of prospective well-being. It is possible and reasonable to make such predictions and anticipate the effects of certain actions to some degree (among others, classical risk assessment as well as cost/benefit analysis offer well-theorized methodology for such endeavors). It is coherent, on my account, to reasonably assume that certain actions are going to objectively and/or subjectively worsen a future person’s well-being, which of course carries normative weight.^22^ It is consequently also possible to say that an event is likely going to cause harm to a future person. Whether or not this circumstance will make their life *not worth living*, however, is a different question, that, as argued above, does not meaningfully contribute to the normative landscape in question.

#### Inaccessibility of subjective quality evaluation to determine LNWL

Given the outlined problems of objective approaches to LNWL, it would seem sensical to turn to subjective approaches instead. However, in beginning-of-life ethics, there is no subject yet who experiences mental states, hence, subjectivity is absent. An applicable subjective approach needs a (present) subject of consideration, which is not the case there. My principal critique of how subjective evaluation approaches are used to attest LNWL thus concerns the issue that subjective perspective on life-worth or preferences for death are not presently obtainable in beginning-of-life cases. Even though this observation may seem trivial, its implications for LNWL determinability are not; they extend towards combined approaches and further lower the reliability of judgments.

The main implication is that when bioethicists talk about the subjective perspective or desire of a possible person whose life is to be assessed as worth living or not, they do not actually refer to *that very person’s* subjective perspective or desire, as the person herself does not exist yet, but the assumption thereof. It thus always relies on an anticipatory evaluation, even when implemented language suggests otherwise (see e.g. Purdy 1996, p.45). As pointed out in the previous section, this criticism does not imply that nothing meaningful can be assumed about the subjective well-being of a prospective person. However, respective assumptions remain speculations to some degree and cannot claim reliability. While it is possible to make educated predictions about a person’s level of prospective well-being, my position holds that this cannot satisfactorily determine a LNWL in advance of subjectivity having formed. Subjective perspective is viewed as necessary for assessing subjective as well as objective goods. To access subjective perspective, an existing subject’s communication (which need not be verbal) is a necessary condition for more reliably interpreting^23^ the quality of the subjective experience in question.

The impossible access to subjective perspective in beginning-of-life is glossed over by proponents who are using LNWL as a threshold for triggering moral duties. External evaluation of subjective perspective must not necessarily be a problem as such, as there are many cases in biomedical ethics where *surrogate decision-making* (see Beauchamp and Childress 2019, ch.3) is the best available option and thus morally justified for the sake of pragmatics. Examples include unconscious patients, infant care, and pediatrics. In the LNWL-implementing beginning-of-life literature, counter to those cases, the jump to surrogate judgment-making on behalf of a yet non-existent subject is not being transparently addressed. The insoluble problem remains that said unreliability of such assessments in beginning-of-life leads to a weaker grounding for reasons towards choosing one course of action over another, and hence an insufficient base for deriving respective moral duties to apply PGD/GGE.

One might reasonably object at this point that the cases in question here are not subject to the condition of personal subjectivity, because no human person is involved and thus that “such decisions are governed by the ethics of ending the lives of non-persons” (Harris 2003, p.10). I would certainly agree to this. Yet, then especially, we must question whether LNWL is useful to implement in beginning-of-life cases. Nearly all LNWL implementations across the bioethical literature include subjectivity as a defining element, while lacking engagement with the fact that subjectivity, and therefore desires or preferences, cannot be reasonably ascribed to an embryo. This imprecision results in missing justification of surrogate judgment making, thus neglecting the argumentative challenges of subjectivist LNWL implementations in beginning-of-life cases.

#### Bias in clear case exemplars to illustrate LNWL

C*lear case* illustrations are used to establish intersubjective plausibility of LNWL. Readers are thus encouraged to assign at least some normative weight to their affective responses to the descriptions or narrations of flagship conditions, and thus to be convinced that LNWL is, at least in those specific genotypes, unquestionably the case.

The main epistemological problem I identify with this approach is that it does not provide any useful information on how exactly the LNWL assessment is made. The symptom descriptions, e.g. of the cognitive impairments caused by Lesch-Nyhan syndrome, are assumed to causally support the LNWL conclusion, without stating which criteria are applied and tested to arrive at this evaluation, and what theory of well-being is underlying. This makes *clear case* exemplars unproductive for deriving more general determining information on identifying LNWL, and thus the evaluation of other cases. While the *clear case* situations are presented as paradigmatic examples of LNWL, it is suggested that there are more to be found. However, given the lack of inferability to other cases, it is doubtful that this is so. Even if the LNWL assessments were correct in the presented cases, no further applicable methodological terms are given. Hence, in the absence of a solid theoretical grounding, supplementing with *clear case* exemplars merely remains a prompt to fill in the conceptual gaps of LNWL with a likely able-bodied and neurotypical intuition.^24^

The result is that *clear case* exemplars problematically instrumentalize abstract narratives^25^ in the sense that they appeal to a certain genre of selective tragic storytelling that heightens the danger of reverting to stereotypes in our intuitive assessments. The selective focus on medical manual descriptions of how certain genetic variations affect the lives of people constitutes a citation bias that largely excludes the perspective of affected persons and their carers.^26^ The potential of narrative for developing intersubjectively plausible ethical assessments thus remains underdeveloped. This is worthy of critique because an argumentative demand for intersubjectivity entails that we do not only consider certain subjects in our efforts to be convincing, but address and cite inclusively.^27^ It is thus important to make a critical effort to engage with all sorts of stories, especially those told by members of marginalized communities (see e.g. Wong 2020). This is needed to avoid the implication that only able-bodied and able-minded people in the medical profession were competent to offer opinions on the matter.^28^

The other critique in this line regards the exclusionary and degrading potential of explicitly ableist language frequently used in *clear case* exemplars. One such example of depicting congenital disability as inherently risky and tragic through metaphoric plot and gripping vocabulary is the following excerpt:

[I]n electing to implant a ‘doomed embryo’, a course of events is set in motion which will result in a predictable future harm, just as surely as when the broken glass is carelessly discarded in the woods. The harm in question, though, will not be a cut foot, but rather a life of such wretched quality that, from the subjective perspective of the child itself, it would have been better to never have been born. (Gavaghan 2007, p.92)

Such stylistic choices of language appeal to a storytelling that heavily carries ableist value judgments and evokes a sense of catastrophe and urgency. Terming potential and actual lives “very terrible” (Glover 2006, p.52), “wretched” (Gavaghan 2007, p.92), “doomed” (Gavaghan 2007, p.92), “burdensome” (Buchanan et al. 2000, p.224), and “miserable” (Steinbock and McClamrock 1994, p.17; Purdy 1996, p.45; Archard 2004, p.408; Singer 2011[1980], p.162), especially without analytically clarifying such terms, does not serve to support unbiased arguments. Participation in public discourse is hindered when preconceived biases already manifest in the language of discussion that is used.

Qualitative inquiry on prospective parents’ experiences with medical providers after receiving a prenatal diagnosis points to ableist language being problematic when used in clinical practice as well (see Koogler et al. 2003). Guon et al. (2014) note that:

One of the most prevalent messages from parental responses is that the language used by providers matters. Terms to describe one’s child such as ‘lethal anomaly’ and statements that the child is ‘incompatible with life’ were often discussed as the least helpful comments made by providers. Comments about lethality may turn normative judgments into clinical ones.” (p.316)

They further emphasize in their clinical guidelines for providers to “[a]void value-laden language related to disability” (Guon et al. 2014, p.316). What kind of vocabulary will be deemed off-limits by this standard, and where the lines lie, cannot be sufficiently explored in this paper. It shall suffice to suggest a mix of commonsense alongside active and broad engagement with widely available disability rights and disability justice advocacy media.

## Discussion: The limited usefulness of LNWL

The preceding analysis has pointed toward significant epistemological and conceptual problems of LNWL for the beginning-of-life context. The relevant task now lies in evaluating whether there could still be usefulness in implementing the concept.

I have provided a case for the secure (enough) determinacy of LNWL not being given in beginning-of-life cases, mainly due to the non-universality of objective approaches, the inaccessibility of subjective perspective, and the risk of misleading stereotyping intuitions appealed by *clear case* exemplars. This indeterminacy is argued to be analogous in LNWL-cognate concepts that formulate higher thresholds for minimal life quality above the LNWL-defining *zero-line*, such as Purdy’s (1996) *minimally satisfying life* or Steinbock’s (2009) *minimally decent life*.

Based on the previously presented arguments, I suggest two independent claims, namely that:

1. a solely objective conception of LNWL is problematic, and
2. LNWL and cognate concepts rest on too insecure a foundation to reasonably support what they are often acclaimed to do: a moral obligation to apply GGE and PGD.

While LNWL is often conceived and used as a reason-giving concept (providing reasons against having a certain child), not all authors who rest their bioethical evaluations of reproductive responsibilities on LNWL/cognate concepts derive a moral duty/obligation/requirement (an example being Glover 2006). However, quite some authors do (e.g. Steinbock and McClamrock 1994; Purdy 1996; Roberts 1998; Buchanan et al. 2000; Benatar 2007; Archard 2004; Harris 2007; Gavaghan 2007).

As claim (1) suggests, an acclaimed moral duty is not a necessary condition for my argument that LNWL is a problematic concept to implement, even if it is used for purely descriptive purposes. Thus, my critique still applies to supposed positions that purport LNWL to not provide prospective parents with decisive reasons not to have a child, and therefore no moral obligation following. This is because my primary critique is of an objective conception of LNWL, which, as detailed previously,^29^ is not reliable. My secondary critique on missing subjectivity and bias equally applies to claim (1).

Regarding the implementation of LNWL as reason-giving, claim (2) suggests that strong reasons that can be reliably determined in a particular case scenario are required to limit reproductive freedom on moral grounds. Such reasons cannot be provided by a concept that rests on epistemically inaccessible presuppositions, as is argued LNWL does in beginning-of-life contexts.

It is imaginable that a more epistemologically humble conception of prospective well-being might be useful to a limited degree to determine morally *permissible* applications of PGD and/or GGE. Yet, given the conceptual shortcomings of LNWL/cognate concepts that I have laid out, the specificities of the conditions for said permissibility have to be still refined: (1) the concepts have to be designed more analytically sturdy (i.e. transparently anchored in established theories of well-being), and (2) a threshold of *high-probability* needs to be clarified. If these issues are sufficiently resolved, educated predictions on prospective well-being could provide a guideline for identifying possible reasons against having a child with a certain genome, while the option to decide to have the child in question remains equally morally defensible.

This also means that prospective parents are not doing something morally wrong if they decide against having a certain child based on its assumed level of future well-being. It is completely rational to make decisions based on the intention of providing one’s child with a good life, and correspondingly taking probability assessments into account, even if the latter are oftentimes (by necessity) vague. Parents are also not committing a moral error even if they decide on the assumption that the future child would lead a LNWL. The case remains that there is no moral wrong when deciding not to have the respective child, even if an objective conception of LNWL (assuming its determinability) is unreliable and remains problematic. It can at the same time be acknowledged that individual decisions based on the LNWL axiom are biased, and that they can (accumulatively) contribute to the preservation of a discursive tendency of de-valuing non-normative bodies. 

Under the premises that underlie liberal-democratic societies, it is possible, and arguably necessary, to tolerate the reproductive choices of individual decision-makers so long as they are not morally wrong. Following my critique, we do not have the epistemological and conceptual grounding to argue that bringing some lives into existence, those thought of as future LNWL, is morally wrong and necessitates a corresponding duty to avoid it.

Tolerance towards such decisions neither necessitates active endorsement of nor opposition to the choices of individuals. The normative content of my paper states that for the sake of conceptual clarity and consistency, and contrary to a popular bioethical opinion, said tolerance must extend towards cases of parents deciding to carry to term an embryo/fetus that is intuitively thought to have a future life not worth living. This is because, according to my argument, the secure-enough anticipation of a LNWL is not epistemologically sound.

Besides epistemological and analytical reasons that point to the limited scope of objective conceptualizations of LNWL in the normativity of beginning-of-life questions, I have suggested that there might also be sociocultural considerations to back this endeavor. A number of key authors in the academic discussion have emphasized the need for a broad societal debate and even a societal consensus before moving forward in developing and applying genetic modification technology in assisted reproduction for humans (see e.g. Lanphier et al. 2015; Lander 2015; Jasanoff et al. 2015; Baylis 2019; Deutscher Ethikrat 2019). Representatives of the disabled community are rightfully pointing out that people with disabilities are not being included in this societal debate nearly as much as they should be. Attorney and legal director of the *Canadian Autistic Self Advocacy Network* Larkin Taylor-Parker recently stated that debating whether disabled lives are worthwhile is usually where discussions on GGE get stuck in practice and do not move to more substantial matters; they call it “the threshold question” (Taylor-Parker 2022, 21:47 min). They say: “To have meaningful conversations about things like heritable genome editing we need to get past that threshold question and recognize that many kinds of human life are worthwhile” (Taylor-Parker 2022, 22:38 min).

Given that LNWL is inherently based off such “ill-posed axiological questions” (Fumagalli 2018, p.789), it also thus adds to the inaccessibility and dysfunctionality of the debate for disabled people. As some authors have already acknowledged, the volume of actual cases where the assumption of a future LNWL might be appropriate is very small in practice (see e.g. Glover 2006; Bennett 2009, 2014; Ranisch 2021). Drastically acknowledging the very limited scope of LNWL and cognate concepts could thus also benefit the public discussion through avoiding to further exclude those who much of the conversation is about.

## Conclusion

This paper has argued that (1) a purely objective conception of LNWL is problematic, and (2) LNWL and cognate concepts are lacking a strong-enough epistemological and conceptual foundation to defend moral reproductive duties. The three common proposals in the literature regarding how LNWL is identifiable have been shown to be unsatisfactory in reliably determining LNWL in beginning-of-life. This has been traced mainly to the incorrect universalization of lived experience by objective approaches, the inaccessibility of subjective perspective, as well as the risk of reverting to stereotyping intuitions through biased language and citation practices in *clear case* exemplars.

Besides epistemological and analytical criticisms that point to problems of LNWL in the normativity of beginning-of-life questions, this paper has presented some sociocultural considerations as well. In order to secure better inclusivity of people with disabilities in the societal debate on GGE and PGD, it has been implied that moving away from a justificatory standard of objective minimal *life-quality/worth* might be beneficial. Instead, the discussion could be opened towards more fruitful qualitative parameters that are developed by and with people with disabilities and thus account for diverse lived experiences. Furthermore, organizers of spaces for relevant public discussion are called to take more proactive measures for designing debates accessibly.^30^ To name just a few examples, wheelchair-supporting venues, audio captioning, sign language interpreters, plain language translations, etc. are needed to establish a societal debate on GGE that includes all members in its decision-making.

There is much more discussion to be had about the future of genetic prediction tools as well as gene editing technologies. Normatively significant reasons might exist for funding GGE research, or even for the justified permissible application thereof in certain instances. This paper has argued that LNWL and cognate concepts, however, are not reliable to decisively establish such reasons. Given the required strength and reliability of reasons to potentially limit reproductive freedom on moral grounds, I am skeptical that a moral duty to apply PGD and/or GGE could ever be reasonably derived. Tentatively, we might thus also extract that the current prioritization of research on the in-vivo delivery of gene editing tools to further somatic applications is appropriate (see e.g. Doudna 2023). The goal of this paper by pointing to the limitations of using LNWL in beginning-of-life has been to sensitize the bioethical discourse to the danger of re-creating ableist biases by formulating moral duties on the conceptually weak basis of LNWL. To offer a suggestion for further research in this realm, we might be well advised to include more insights from qualitative empirical fieldwork on people’s lived experiences with certain genetic conditions as well as to increase involvement of said community stakeholders in normative research designs.

## Footnotes

1 A question that certainly arises in this context is the one of sameness and/or difference of PGD versus GGE interventions. The difference is certainly practical (as in different technologies) as well as philosophical (genetic selection versus alteration, see e.g. Ranisch 2019). However, this difference is not essential to my argument here. Thus, the differences between PGD and GGE interventions shall not be explored in detail in this paper and the focus is laid on LNWL-based justifications/requirements thereof.

2 A contentious question is whether a distinctly different child would be created through an alteration via implementing GGE, so to say, whether GGE is identity-affecting or person-affecting. This also touches on the question of whether GGE can be argued to be therapeutic or not, and if embryos can be framed as patients. See more on this question in e.g. Cavaliere (2018).

3 On the idea of coming into existence as harm see Benatar (2007).

4 Sometimes the concept is also termed ‘wrongful life’ in the literature. So to not get confused with legal definitions of the concept, the term ‘life not worth living’/LNWL is used here throughout. Excluded from this discussion are legal notions of the idea manifesting in ‘wrongful life’ or ‘wrongful birth’ civil claims and resulting lawsuits in certain countries’ legislations such as the Netherlands, the UK, South Africa, or the US (see ten Have and do Céu Patrão Neves 2021).

5 ‘LNWL’ is used as an umbrella term for both in the following, ‘and cognate concepts’ is only occasionally spelled out. Respective differences are discussed in Sect. “Discussion: The limited usefulness of LNWL”.

6 It is important to note that the scrutinized arguments are neither homogenous in their normative conclusions, nor can they be said to represent a canonical/standard view of the field. The presented scholars diverge in their individual arguments quite a bit at times, and while LNWL is present, it is not always the primary constituent of an oftentimes overall larger claim. What the scrutinized texts share is the implementation of LNWL as well as the property of being widely referenced works in the field.

7 End-of-life cases present a very different situation, e.g. an existing person themselves deciding that they no longer find their lives worth living and would prefer to be assisted to die (see e.g. Schaber 2017). My conceptual and epistemological criticism on LNWL does not necessarily extend to those cases, granted that subjectivity exists and thus a self-assessment condition along with the criterion of informed consent can be fulfilled (see Harris 2003; Peled et al. 2017).

8 Relevant bioethical institutions and stakeholders such as the *Nuffield Council*, the *German Ethics Council*, the *UK Academy of Medical Sciences*, *US National Academies of Sciences and Medicine* and *World Academy of Sciences* and the *Royal Society* and *National Academies* (the latter co-organizing the *International Summits on Human Heritable Genome Editing*) have an overt interest in providing public access to their work and events on ARTs as well as disseminating their recommendations to a larger audience to foster public societal debate.

9 An exception to this is Glover (1977, 2006) who dedicates a lot of thought and analytic exploration to the conceptual intricacies of LNWL.

10 On objective list theories and discussion thereof see e.g. Finnis (1980), Parfit (1976, 1984), and Fletcher (2013); see also the influential *capabilities*-approach developed by Nussbaum (1999, 2006, 2011).

11 Informed preference is commonly viewed as a special subgroup of desire fulfillment theories. In the case of beginning-of-life, this informed preference is constituted by assuming the prospective person in question would prefer what is rationally best for them (see e.g. Sobel 1994). On rational desire and LNWL see also Glover (1977).

12 A much debated, extreme version of the *worse-than-nonexistence* view is defended by Benatar (2007). Other common comparisons refer to e.g. *most people’s lives* (e.g. Buchanan et al. 2000, p.224).

13 On the difference between those conceptions and the differing corresponding thresholds see Ranisch (2021, pp.248–255).

14 On the shared properties of these subjectivist approaches see Heathwood (2006).

15 See e.g. on missing awareness for researcher positionality in qualitative research Oakley (1981); Anandhi and Velayudhan (2010); Griffin (2012); on intersectional critiques of feminist epistemology see Collins (1990), hooks (2015), Kendall (2020). On the moral problems of “you say you’re happy, but…” arguments regarding the lives of disabled people see Goering (2008) as well as Marsh (2019). On the discursive exclusion of people with disabilities see also e.g. Charlton (2000); Kafer (2013); Taylor (2018); Piepzna-Samarasinha (2018).

16 *Ableism*, adjective *ableist*, according to disability *justice* advocate Alice Wong, refers to “a form of oppression that systematically devalues disabled people who are considered non-normative in the way they look, behave, move, think, or their way of being in the world” (Wong 2017).

17 The alternative *social model* has thus, in contrast to the medical model, proposed the distinction between disability (located outside the body as environmental barriers) and impairment (as located inside the body, e.g. pain, physiological symptoms). Contemporarily, this model is increasingly challenged for being too binary and simplistic; yet it is still used regularly for its advantages of structural clarity, see e.g. Shakespeare (2013). It is important to acknowledge that some authors do indeed make an effort to factor in environmental and social aspects into LNWL judgments, e.g. Wilkinson (2010) and Archard (2004).

18 Empirical research also points to differing judgments of respective levels of life quality of people with disabilities compared to people without disabilities, see Albrecht and Devlieger (1999); Rand and Caiels (2015).

19 One could hypothesize that this kind of clear-cut judgment might be appropriate for cases where the life contains a total of zero benefits compared to harms, hence lives of *pure harm*. Yet, such cases are largely philosopher’s abstractions, with a low likelihood of matching any corresponding real-life cases.

20 In addition, how much pain, how frequent, how short the life expectancy etc. is not specified.

21 So, when exactly we would have decisive moral reasons to not reproduce or to create a different child than we would otherwise have had.

22 This is also the case regarding far future people, taking for instance the normative significance of anticipated effects of climate change on future generations. The author thanks an anonymous reviewer for this consequential extension of the presented argument.

23 Naturally, such interpretations can be false. Hence, they also come with a degree of unreliability, especially when verbal communication is absent. This unreliability equally exists regarding subjects who do communicate verbally, since it is possible to lie about subjective well-being. However, I would argue that interpreting verbal as well as non-verbal communication according to social and cultural codes is not quite as unreliable as anticipating a yet non-existent subject’s mental states.

24 On prevailing anti-disability bias see e.g. Albrecht and Devlieger (1999); Wilson and Scior (2014); Kaposy (2019).

25 Abstract narratives in the sense that the quoted passages, while including narrative elements and devices, rarely represent a fully-fledged plot. A few examples of narrative devices in clear case presentations are e.g. Purdy (1996), quoting a gripping entry statement from *The Merck Manual* (1972, pp.1363, 1346) “Onset is insidious”, or the strategic parallelism “walking becomes impossible, swallowing difficult, and dementia profound”.

26 Although some authors make an effort to also cite people who live with or are in close contact with the flagship conditions they use, it is not very common in most of the LNWL literature. For example, Purdy (1996, p.44) gives a brief citation of a person with a congenital condition, yet only to present them as a defender of a polarized optimistic position in the debate that is almost caricatured as naive. This kind of citation practice does not seem to further an inclusive and differentiated debate, either.

27 This means quoting and involving in normative research designs all sorts of people, including affected persons, caregivers, disability rights and justice advocates, and so on. Further steps could include translating bioethical texts into plain language to publish alongside original academic articles, and to ensure audio captions or sign language translations are provided at relevant public events for accessibility of the debate.

28 For more on epistemic humility regarding GGE applications see Garland-Thomson (2020).

29 See Sect. “Principal problems with objective quality evaluation to determine LNWL”.

30 For instance, the National Academies of Sciences, Engineering, and Medicine that hosts the *International Summits on Human Gene Editing* did not plan on providing audio captions for the public online stream of the event until Teresa Blankmeyer Burke, Professor of philosophy at Gallaudet University and advocate of the deaf community, actively pointed it out to a member of the organizing committee (see Blankmeyer Burke 2022, 39:17 min).
